# The avian-specific small heat shock protein HSP25 is a constitutive protector against environmental stresses during blastoderm dormancy

**DOI:** 10.1038/srep36704

**Published:** 2016-11-09

**Authors:** Young Sun Hwang, Mee Hyun Ko, Young Min Kim, Young Hyun Park, Tamao Ono, Jae Yong Han

**Affiliations:** 1 Department of Agricultural Biotechnology and Research Institute of Agriculture and Life Sciences, College of Agriculture and Life Sciences, Seoul National University, Seoul 08826, Korea; 2 Division of Animal Science, Faculty of Agriculture, Shinshu University, Minamiminowa, Nagano 399-4598, Japan; 3 Institute for Biomedical Sciences, Shinshu University, Minamiminowa, Nagano 399-4598, Japan

## Abstract

Small heat shock proteins (sHSPs) range in size from 12 to 42 kDa and contain an α-crystalline domain. They have been proposed to play roles in the first line of defence against various stresses in an ATP-independent manner. In birds, a newly oviposited blastoderm can survive several weeks in a dormant state in low-temperature storage suggesting that blastoderm cells are basically tolerant of environmental stress. However, sHSPs in the stress-tolerant blastoderm have yet to be investigated. Thus, we characterised the expression and function of sHSPs in the chicken blastoderm. We found that chicken *HSP25* was expressed especially in the blastoderm and was highly upregulated during low-temperature storage. Multiple alignments, phylogenetic trees, and expression in the blastoderms of Japanese quail and zebra finch showed homologues of HSP25 were conserved in other avian species. After knockdown of chicken *HSP25*, the expression of pluripotency marker genes decreased significantly. Furthermore, loss of function studies demonstrated that chicken *HSP25* is associated with anti-apoptotic, anti-oxidant, and pro-autophagic effects in chicken blastoderm cells. Collectively, these results suggest avian *HSP25* could play an important role in association with the first line of cellular defences against environmental stress and the protection of future embryonic cells in the avian blastoderm.

Heat shock protein (HSP) levels increase in response to various cellular stresses and they function as molecular chaperones which bind to and inhibit irreversible protein aggregation or misfolding under stressful conditions^1^. Among HSP families, members of the small heat shock protein (sHSP) family range in size from 12 to 42 kDa, and possess highly variable N-terminal and C-terminal regions and conserved α-crystallin domains^2^. Monomers of sHSP can interact and bind themselves via the α-crystallin domain to form dimers or higher oligomers, assisted by the N- and C-terminal regions. Unlike other HSPs, sHSPs function as holdases in the absence of ATP and can bind various protein substrates, thereby contributing to cell survival^3^. This ATP-independent holdase function of sHSPs is especially important when the ATP concentration is low or limited.

Induction of sHSP expression is stimulated by stress, but may also be under developmental control or regulated in a cell- and tissue-specific manner^4^,^5^. During early gastrulation in *Danio rerio*, *heat shock protein family B (small) member 1* (*hspb1*) gene is expressed transiently in the developing myotome, lens, and presumptive brain^6^. Constitutive expression of the *hsp30* genes has been detected in the cement gland of early and mid-tailbud *Xenopus laevis* embryos^7^. In mouse pre-implantation embryos, *Hspb1* mRNA is induced by zygotic genome activation at the two-cell stage, is subsequently decreased at the four-cell stage, and is re-upregulated at the morula stage, with the highest expression in the blastocyst^8^. Additionally, mouse embryonic stem cells show a unique stress-resistant gene expression signature, including *Hspb1*, that becomes downregulated during embryoid body formation^9^.

In particular, sHSP genes are expressed specifically in the dauer stage of *Caenorhabditis elegans* and in the cyst of *Artemia franciscana*, which are so-called diapause states described in many reports as stress-tolerant and developmental arrest^10^,^11^. The *C. elegans* dauer stage with DAF-16 activity undergoes marked induction of several sHSPs, including *hsp16.1*, *hsp16.49*, *hsp-12.6*, *hsp-12.3*, *hsp-20*, and *sip-1*^12^,^13^. In the case of *A. franciscana*, *p26*, *ArHsp21*, and *ArHsp22* are expressed specifically in diapause, and reach a peak of expression in the cyst, while they are not detected during development^14^,^15^,^16^,^17^. Moreover, a lack of p26 in the cyst resulted in the spontaneous termination of diapause^18^.

Eleven small heat shock protein genes from chicken are entered in the GenBank database, including the HSP30 subfamily, which is restricted to oviparous animals^19^. Chicken sHSPs induced by heat shock and chemical stresses have been studied and observed in somatic fibroblast cells^19^ and adult tissues^20^,^21^,^22^,^23^. In avian species, although embryonic diapause has not been reported, developmental arrest, so-called cold torpor, of the embryos of the earlier eggs in a single clutch is common at the beginning of incubation, until all eggs in the clutch have been laid^24^. Bloom *et al.* reported that chicken Eyal-Giladi and Kochav (EGK) stage X blastoderms after oviposition^25^ could survive 3–4 weeks in a dormant state under conditions of cold storage and that this involved expressing *HSP70* and anti-apoptotic B-cell CLL/lymphoma *(BCL*) *family* genes^26^. However, the expression and functions of small HSPs in the stress-tolerant avian blastoderm have yet to be investigated.

In this study, we first identified the expression and function of sHSPs in the blastoderm to assess which sHSP was associated with the stress-resistant characteristics of blastoderm cells in chickens. We also demonstrated that avian *HSP25* was important in the protection of future embryonic cells in the blastoderm.

## Results

### Expression profiling of sHSPs in chicken stage X blastoderm

**Figure 1:**
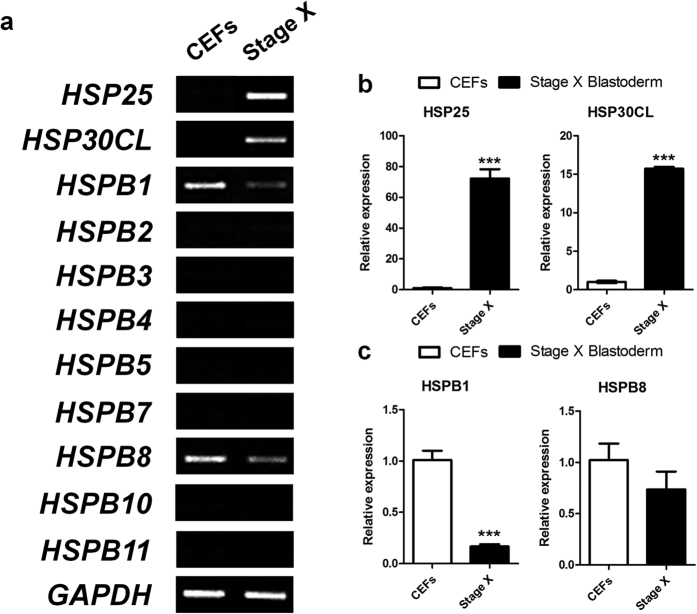
Expression profiling of sHSPs. Expression analysis of *sHSPs* in chicken embryonic fibroblasts (CEFs) and chicken stage X blastoderm (Stage X) by RT-PCR **(a)** and quantitative real-time PCR **(b,c)**. *HSP25* and *HSP30CL* were detected specifically in stage X blastoderm **(b)**. Real-time PCR was conducted in triplicate and normalised to expression of *GAPDH*. Significant differences between groups are indicated as ***P < 0.001. Error bars indicate the SE of triplicate analyses.

To identify the expression of sHSPs in chicken stage X blastoderm, 11 *sHSP* genes from the GenBank database were examined by RT-PCR and qRT-PCR using cDNA from CEFs and stage X blastoderms. *HSP25* and *HSP30CL* were detected specifically in stage X blastoderm. However, *HSPB1* was expressed specifically in CEFs and *HSPB8* was expressed more abundantly in CEFs (Fig. 1a). Other small heat shock genes were not detected in either. Furthermore, qRT-PCR analysis showed that *HSP25* and *HSP30CL* expression levels were 72.2-fold and 15.7-fold higher in stage X blastoderms than CEFs, respectively (Fig. 1b), while *HSPB1* and *HSPB8* expression levels were 6-fold and 1.4-fold lower in stage X blastoderms than CEFs, respectively (Fig. 1c).

### Expression of chicken stage X blastoderm-specific HSP30 subfamily members during intrauterine development, egg incubation, and egg storage

**Figure 2:**
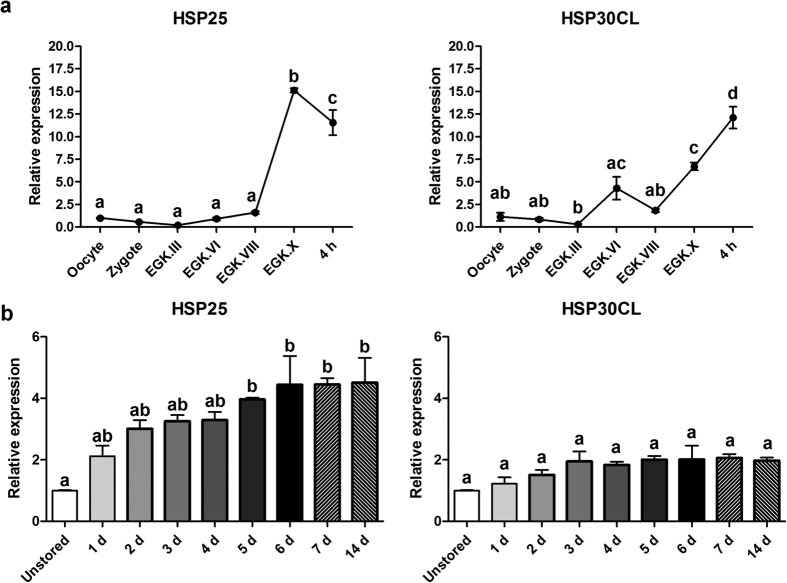
Expression dynamics of chicken stage X blastoderm-specific HSP30 subfamily. Quantitative expression analysis of blastoderm-specific chicken *HSP30 subfamily* during intrauterine and early development **(a)** and egg storage **(b)**. Real-time PCR was conducted in triplicate and normalised to expression of *GAPDH* and *ACTB*. Significant differences between groups are indicated by different letters. Error bars indicate the SE of triplicate analyses. EGK, Eyal-Giladi and Kochav stage; 4 h, 4-h-incubated embryo at 37.5 °C; d, days of storage at 16 °C.

As shown above, chicken *HSP25* and *HSP30CL* were expressed specifically in stage X blastoderms. Next, we investigated the expression patterns during intrauterine and early development and egg storage. First, we examined the expression patterns of the HSP30 subfamily during chicken intrauterine development. Expression of both genes was detected at low levels until EGK. III at which point zygotic gene activation (ZGA) occurred. In the case of *HSP25*, after EGK.VIII, expression increased sharply only shortly before oviposition, whereas, *HSP30CL* expression increased after EGK.III and EGK.VIII (Fig. 2a). Furthermore, after a 4 h incubation of eggs at 37.5 °C to promote embryonic development to the pre-streak stage^25^, expression of *HSP25* decreased significantly. However, *HSP30CL* expression in the 4-h-incubated embryo was significantly higher than in stage X blastoderm (Fig. 2a). To examine *HSP30* subfamily expression dynamics due to increased stress in the stage X blastoderm, we also quantified transcripts during egg storage at 16 °C. Expression of both *HSP25* and *HSP30CL* was upregulated gradually during egg storage. However, *HSP25* mRNA expression increased significantly after 5 days of storage versus the unstored blastoderm and this was maintained until 14 days, whereas upregulation of *HSP30CL* mRNA expression was not significant (Fig. 2b).

### Multiple sequence alignment, phylogenetic analysis, and expression in other avian blastoderms of sHSPs containing the α-crystallin domain HSPB9-like

**Figure 3:**
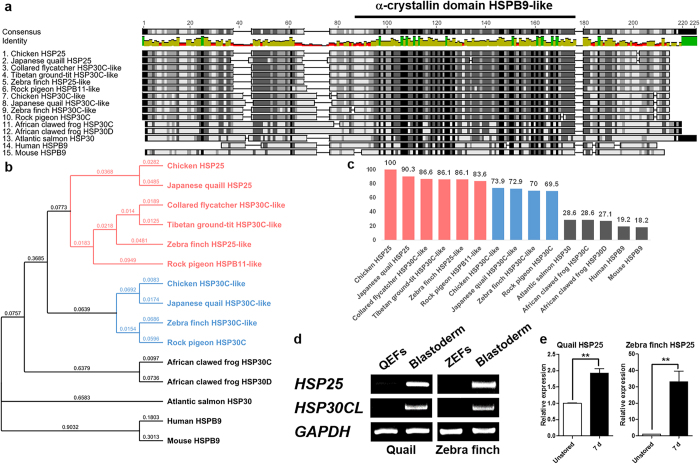
Multiple alignment and phylogenetic trees of sHSPs containing the α-crystallin domain HSPB9-like. Multiple sequence alignment **(a)**, phylogenetic trees **(b)**, and similarity to chicken HSP25 **(c)** of the amino acid sequences of sHSPs containing the α-crystallin domain HSPB9-like. Bold lined amino acids indicate the α-crystallin domain HSPB9-like. Avian HSP25 in red and HSP30CL in blue. Expression analysis of *HSP25* and *HSP30CL* in quail and zebra finch by RT-PCR **(d)** and during egg storage by qRT-PCR **(e)**. Real-time PCR was conducted in triplicate and normalised to expression of *GAPDH*. Significant differences between groups are indicated as **P < 0.01. Error bars indicate the SE of triplicate analyses. QEFs, quail embryonic fibroblasts; ZEF, zebra finch embryonic fibroblasts; Blastoderm, blastoderm at oviposition in each species; d, days of storage at 16 °C.

mRNA and protein sequences of chicken HSP25 were obtained from the NCBI *Gallus gallus* genome database. The *HSP25* mRNA sequence contains an open reading frame of 582 base pairs with no intron sequence and encodes 194 amino acids, including the α-crystallin domain HSPB9-like. Next, we aligned the protein sequences containing the α-crystallin domain HSPB9-like from chicken HSP25, HSP30C-like, Japanese quail HSP25, HSP30C-like, collared flycatcher HSP30C-like, Tibetan ground-tit HSP30C-like, zebra finch HSP25-like, HSP30C-like, rock pigeon HSPB11-like, HSP30C, African clawed frog HSP30C, HSP30D Atlantic salmon HSP30, human HSPB9, and mouse HSPB9 (Fig. 3a). Pair-wise comparisons of sHSPs showed high degrees of homology among avian species including the α-crystallin domain, but they differed from other vertebrate sequences. Also, in the phylogenetic analysis, and the degree of similarity to chicken HSP25, they separated into two subfamilies, forming a branch separated from other vertebrates, one including chicken HSP25, Japanese quail HSP25, collared flycatcher HSP30C-like, Tibetan ground-tit HSP30C-like, zebra finch HSP25-like, and rock pigeon HSPB11-like, and another including chicken HSP30C-like, Japanese quail HSP30C-like, zebra finch HSP30C-like, and pigeon HSP30C due to N-terminal variations between the subfamilies (Fig. 3b,c). To identify the expression patterns of *HSP25* and *HSP30CL* in other avian species, we compared QEFs, ZEFs, and blastoderms at oviposition by RT-PCR. Similar to chicken, expression of the Japanese quail and zebra finch *HSP25* and *HSP30CL* genes was detected specifically in blastoderms (Fig. 3d). Furthermore, qRT-PCR analysis showed that *HSP25* mRNA expression increased significantly after 7 days of storage versus the unstored blastoderm in quail and zebra finch (Fig. 3e), similar to the case of chicken embryo.

### Effect of *HSP25* knockdown in chicken blastoderm cells

**Figure 4:**
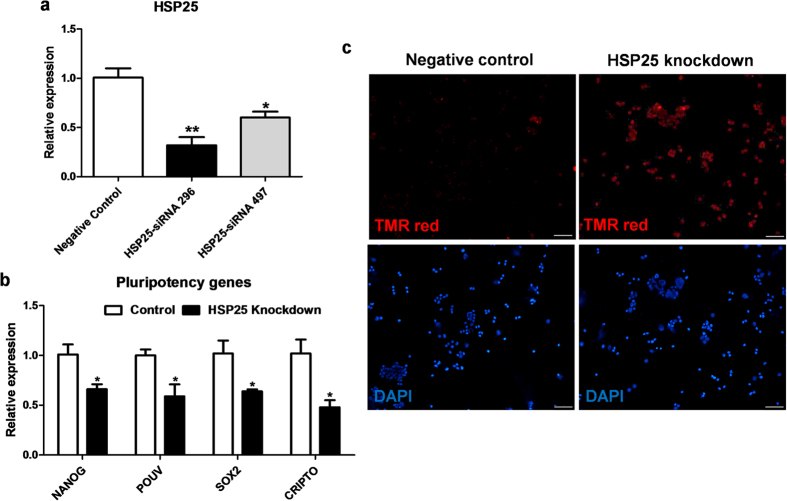
Knockdown analysis of HSP25 in chicken blastoderm cells. **(a)** Knockdown efficiency of *HSP25*-specific siRNAs in chicken blastoderm cells. Non-complementary sequences in the chicken genome were used as a control. **(b)** Relative expression analysis of pluripotency marker genes after *HSP25* knockdown. Real-time PCR was conducted in triplicate and normalised to the expression of *GAPDH*. Significant differences between groups are indicated as **P < 0.01 and *P < 0.05. Error bars indicate the SE of triplicate analyses. **(c)** TUNEL assay performed on chicken blastoderm cells after *HSP25* knockdown. Scale bars are 50 μm.

To investigate chicken *HSP25* function, we designed two candidate siRNAs, siRNA-296 and siRNA-497, and transfected them into blastoderm cells *in vitro*. At 48 h after transfection, the *HSP25* transcript level was analysed using real-time PCR. As shown in Fig. 4a, siRNA-296 was the most efficient knockdown probe for *HSP25*, showing 68% suppression versus the control (*P* < 0.01). This siRNA was selected for further knockdown experiments of chicken *HSP25*. First of all, we examined the role of chicken *HSP25* in stem cell property, like as mouse ESCs^5^,^9^. After knockdown of *HSP25* in chicken blastoderm cells, we examined the expression of pluripotency markers, including *nanog homeobox* (*NANOG*), *POU domain class 5 transcription factor 3* (*POUV*), *SRY (sex determining region Y)-box 2* (*SOX2*), and *cripto, FRL-1, cryptic family 1B* (*CRIPTO*). Compared with the control, knockdown of *HSP25* in blastoderm cells decreased the expression of all pluripotency genes significantly (*P* < 0.05; Fig. 4b). Furthermore, we performed a TUNEL assay to examine the effects of *HSP25* knockdown on apoptosis in chicken blastoderm cells. Apoptotic signals were not detected in the control samples, but were strongly induced in most blastoderm cells after *HSP25* knockdown (Fig. 4c).

### Anti-apoptotic function of *HSP25* in chicken blastoderm cells

**Figure 5:**
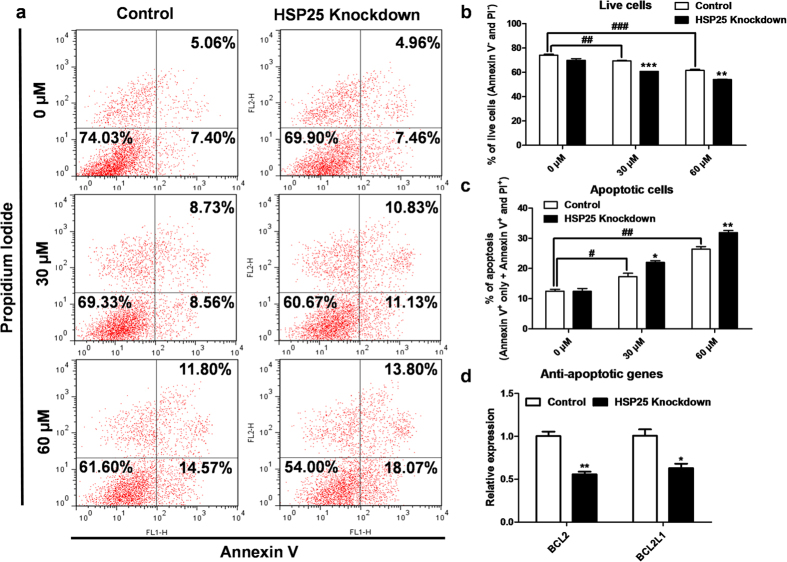
Anti-apoptotic function of HSP25 in mitomycin C-treated chicken blastoderm cells. **(a)** Annexin V/PI analysis by flow cytometry after *HSP25* knockdown followed by mitomycin C treatment (0, 30, or 60 μM). Quantitative analysis of double-negative **(b)** and double-positive **(c)** cells, indicating live and apoptotic cells, respectively. **(d)** Relative expression analysis of anti-apoptotic genes after *HSP25* knockdown followed by 60 μM mitomycin C treatment. Non-complementary sequences in the chicken genome were used as a control. Real-time PCR was conducted in triplicate and normalised to expression of *GAPDH*. ^###^*P* < 0.001 and ^##^*P* < 0.01 significance of mitomycin C treatment (30, or 60 μM) compared to 0 μM. Significant differences between control and *HSP25* knockdown are indicated as ***P < 0.001, **P < 0.01, and *P < 0.05. Error bars indicate the SE of triplicate analyses.

Next, we investigated chicken *HSP25* function in blastoderm cell survival under various stress conditions. First, to study the anti-apoptotic function of chicken *HSP25*, we assessed apoptotic cell death in blastoderm cells after *HSP25* knockdown followed by mitomycin C treatment with annexin V/PI and flow cytometry analysis (Fig. 5a). After mitomycin C treatment on blastoderm cells, the proportion of live cells was decreased (annexin V^−^ and PI^−^, Fig. 5b) and apoptotic cells (annexin V^+^ only + annexin V^+^ and PI^+^, Fig. 5c) was increased significantly. Then, compared with the control, *HSP25* knockdown decreased the proportion of live cells significantly when treated with mitomycin C at 30 μM and 60 μM (*P* < 0.001 and < 0.01, respectively; Fig. 5b). Also, Fig. 5c shows that the repression of *HSP25* increased the proportion of apoptotic cells significantly (30 μM, *P* < 0.05 and 60 μM, *P* < 0.01). Correspondingly, expression of anti-apoptotic genes, such as *BCL2* and *BCL2L1*, was decreased significantly by *HSP25* knockdown followed by 60 μM mitomycin C treatment in chicken blastoderm cells (Fig. 5d).

### Anti-oxidant and anti-necrotic effects of *HSP25* in chicken blastoderm cells

**Figure 6:**
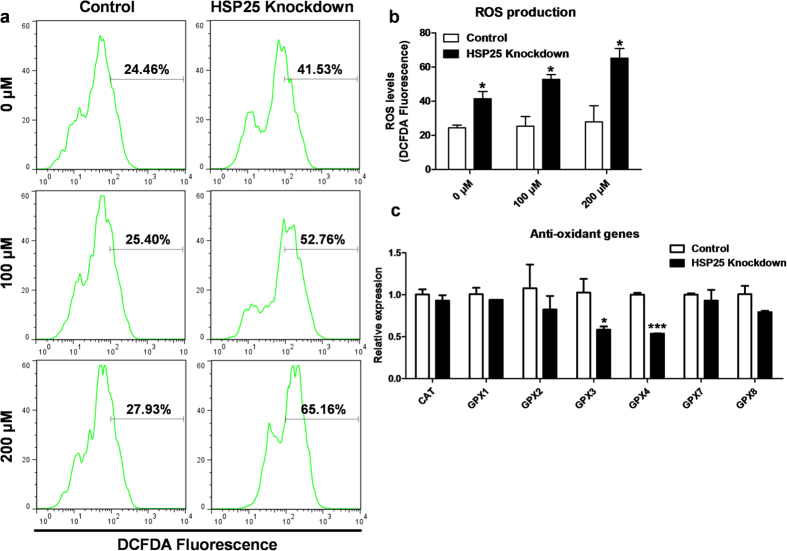
Anti-oxidant effect of HSP25 in H_2_O_2_-treated chicken blastoderm cells. **(a)** DCFDA analysis by flow cytometry after *HSP25* knockdown followed by H_2_O_2_ treatment (0, 100, or 200 μM). **(b)** Quantitative analysis of DCFDA-positive cells. **(c)** Relative expression analysis of anti-oxidant genes after HSP25 knockdown followed by 200 μM H_2_O_2_ treatment. Non-complementary sequences in the chicken genome were used as a control. Real-time PCR was conducted in triplicate and normalised to expression of *GAPDH*. Significant differences between control and *HSP25* knockdown are indicated as ***P < 0.001 and *P < 0.05. Error bars indicate the SE of triplicate analyses.

Chicken blastoderm cells were treated with H_2_O_2_ to create oxidative stress after *HSP25* knockdown, then a DCFDA assay was performed with flow cytometry to measure ROS in blastoderm cells (Fig. 6a). The DCFDA analysis showed that ROS production was significantly higher after *HSP25* knockdown than the control with or without H_2_O_2_ treatment (*P* < 0.05; Fig. 6b). Furthermore, we conducted a gene expression analysis of anti-oxidant genes by real-time PCR. Figure 6c shows that two anti-oxidant genes, *glutathione peroxidase 3* (*GPX3*) and *glutathione peroxidase 4* (*GPX4*), were downregulated after *HSP25* knockdown followed by 200 μM H_2_O_2_ treatment.

**Figure 7:**
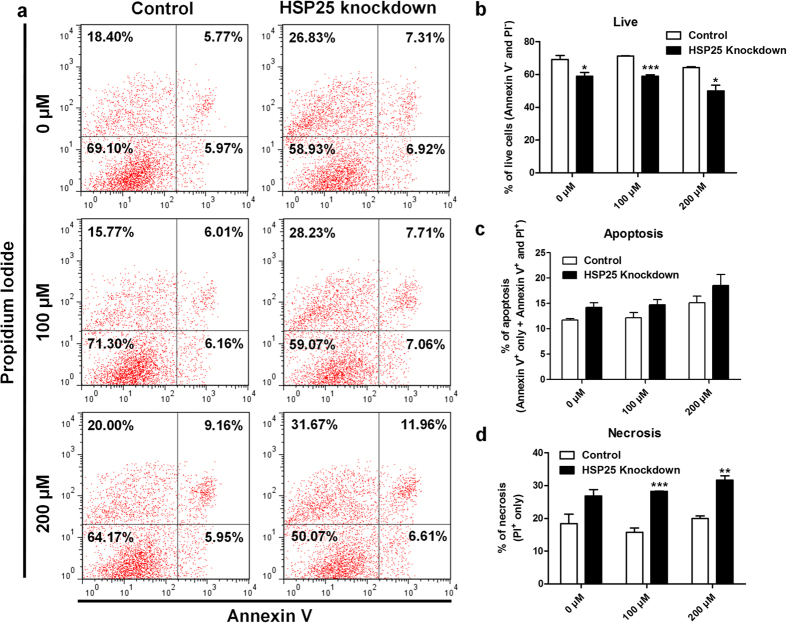
Anti-necrotic effect of HSP25 in H_2_O_2_-treated chicken blastoderm cells. **(a)** Annexin V/PI analysis by flow cytometry after *HSP25* knockdown followed by H_2_O_2_ treatment (0, 100, or 200 μM). Quantitative analysis of double-negative **(b)**, double-positive **(c)** and PI-positive only **(d)** cells, indicating live, apoptotic, and necrotic cells, respectively. Non-complementary sequences in the chicken genome were used as a control. Significant differences between control and *HSP25* knockdown are indicated as ***P < 0.001, **P < 0.01, and *P < 0.05. Error bars indicate the SE of triplicate analyses.

Generally, oxidative stress due to H_2_O_2_ promotes cell death in various cell types. Thus, we also used annexin V/PI and flow cytometry to investigate effects of *HSP25* on cell death with oxidative stress (Fig. 7a). After knockdown of *HSP25*, double negative annexin V/PI cells (live cells; Fig. 7b) were decreased significantly with H_2_O_2_ treatment (100 μM, *P* < 0.01 and 200 μM, *P* < 0.05). Notably, although the proportion of apoptotic cells (annexin V^+^ only + annexin V^+^ and PI^+^) was slightly, but not significantly increased (Fig. 7b), that of necrotic cells (PI^+^ only) was increased significantly after *HSP25* knockdown followed by H_2_O_2_ treatment (100 μM, *P* < 0.001 and 200 μM, *P* < 0.01; Fig. 7c).

### Pro-autophagic function of *HSP25* in chicken blastoderm cells

**Figure 8:**
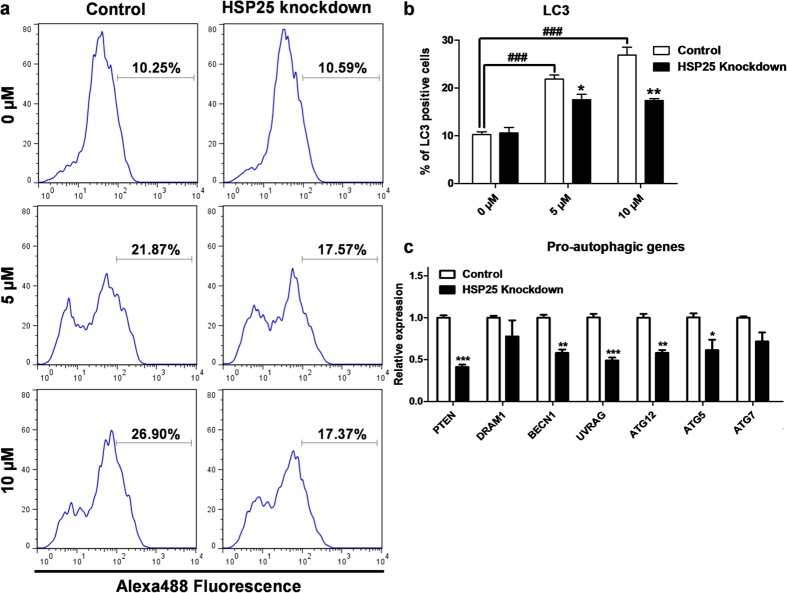
Pro-autophagic effect of HSP25 in MG132-treated chicken blastoderm cells. **(a)** Flow cytometry analysis of blastoderm cells with LC3 after *HSP25* knockdown followed by MG132 treatment (0, 5, or 10 μM). An Alexa488-conjugated secondary antibody for rabbit IgG was used. **(b)** Quantitative analysis of LC3-positive cells. **(c)** Relative expression analysis of pro-autophagic genes after HSP25 knockdown followed by 10 μM MG132 treatment. Non-complementary sequences in the chicken genome were used as a control. Real-time PCR was conducted in triplicate and normalised to expression of *GAPDH*. ^###^*P* < 0.001 significance of MG132 treatment (5, or 10 μM) compared to 0 μM. Significant differences between control and *HSP25* knockdown are indicated as ***P < 0.001, **P < 0.01, and *P < 0.05. Error bars indicate the SE of triplicate analyses.

We examined the pro-autophagic function of chicken *HSP25* in blastoderm cells by siRNA knockdown and autophagy stimulation experiments. After 48 h of knockdown of chicken *HSP25*, we treated cells with MG132 at 5 and 10 μM to induce autophagy, then analysed the cells using flow cytometry for LC3 antibody-Alexa488 fluorescence (Fig. 8a). After MG132 treatment on blastoderm cells, the stimulation of autophagy was increased significantly (Fig. 8b). Then, quantification of LC3-positive cells showed that *HSP25* knockdown decreased autophagy activation significantly under MG132 induction versus the control. However, there was no difference between knockdown and the control without MG132 induction (Fig. 8b). Real-time PCR analysis of pro-autophagic genes was also performed. Figure 8c shows that expression levels of several pro-autophagic genes, such as *phosphatase and tensin homolog* (*PTEN*), *beclin 1, autophagy related* (*BECN1*), *UV radiation resistance associated* (*UVRAG*), *autophagy related 12* (*ATG12*), and *autophagy related 5* (*ATG5*), were downregulated after *HSP25* knockdown followed by 10 μM MG132-induced autophagy.

## Discussion

sHSPs are ATP-independent, contain an α-crystallin domain, and prevent the irreversible denaturation of other proteins. sHSPs are known to be responsible for the transfer of other proteins to the ATP-dependent chaperones or to the protein degradation machinery, such as proteasomes or autophagosomes^3^. In many organisms, sHSPs are accumulated for stress tolerance during diapause and other dormant states in which the ATP concentration is low or limited^27^,^28^. Among avian species, newly oviposited EGK stage X chicken blastoderms can endure in a dormant state known as cold torpor for 3–4 weeks^26^,^29^. Thus, we hypothesised that chicken blastoderm may show specific sHSP protein expression and function.

First, we examined the expression of 11 *sHSPs*, including the *HSP30* subfamily, in chicken EGK stage X blastoderm cells. Among them, *HSP25* and *HSP30CL* of the HSP30 subfamily were expressed specifically in the chicken blastoderm and *HSP25* showed higher expression than *HSP30CL*. The HSP30 subfamily is absent from mammals and may be restricted to oviparous animals, such as frogs, fish, and avians^19^,^30^,^31^. Thus, we identified two sHSPs which may be candidates for regulating dormancy in the chicken blastoderm.

Next, we analysed the expression patterns of *HSP25* and *HSP30CL* during intrauterine and early development. *HSP25* mRNA was upregulated exclusively after EGK.VIII, shortly before oviposition, whereas *HSP30CL* mRNA was induced after EGK.III, subsequently decreased at EGK.VI, and upregulated again after EGK.VIII. These findings indicated that *HSP25* and *HSP30CL* transcripts were not maternally inherited for embryonic development and stress tolerance. Additionally, developmentally constitutive *HSP30* homologues in *Xenopus* were first detected in early and mid-tailbud embryos, after gastrulation, but not soon after ZGA, which in *Xenopus* occurs at the 128- to 256-cell stage^7^. It was recently discovered that ZGA starts between EGK stages II and III in chicken^32^,^33^. *HSP25* and *HSP30CL* transcripts were also not induced immediately after ZGA, at EGK III, but earlier than the homologues in *Xenopus*. Accordingly, *HSP25* expression may be related to gaining embryonic tolerance against environmental stress; however, the expression pattern of *HSP30CL* may indicate effects at both the cleavage stage, between EGK.III and VI, and later. Additionally, *HSP25* mRNA was decreased significantly, whereas *HSP30CL* mRNA was upregulated significantly when embryonic development was reinitiated in the 4-h-incubated embryo versus the stage X blastoderm. Species-specific *sHSP* gene expression was increased and expressed exclusively in diapause and dormancy states, such as the dauer stage of *C. elegans* and cysts of *A. franciscana*, but was not detected when development continued^10^,^11^. Thus, *HSP25* gene expression was reduced in developmental initiation, but the *HSP30CL* gene was induced, more similar to the gastrulation stages in *Xenopus*^7^.

Next, we examined *HSP25* and *HSP30CL* expression during egg storage, a dormant state in which energy production was limited. Even in this dormant state under cold storage of eggs for 14 days, *HSP25* expression was upregulated significantly by approximately four-fold after 5 days of storage versus unstored blastoderm, and this level was maintained through 14 days of storage. However, the level of *HSP30CL* expression after storage was only about two-fold higher (not significant). As shown in Fig. 1a, *HSP25* transcript levels were the highest of the *sHSPs* in stage X blastoderms. Collectively, these results indicated that, during early development in chicken, *HSP25* was most abundant, and may be specific for embryonic programming of self-defence against future stresses after oviposition.

To identify *HSP25* homologues in vertebrates, we generated multiple alignments and performed phylogenetic tree analysis of sHSPs containing α-crystallin domains HSPB9-like in chicken, Japanese quail, collared flycatcher, Tibetan ground-tit, zebra finch, rock pigeon, frog, salmon, human, and mouse. According to the multiple alignment and phylogenetic trees, the α-crystallin domain in sHSPs was highly conserved, but there was also distinct variations in the N-terminal domain among species. In particular, the N-termini of avian sHSPs differ significantly from those of human, mouse, salmon, and frog sHSPs. Thus, HSP25 and HSP30CL in avian species are quite different to those of other vertebrates.

Although the structure and organisation of the N-terminus in sHSPs is not yet well defined, it is known that the N-terminal domains determine phosphorylation sites, and dimer and oligomer organisation^2^,^3^. Thus, the N-terminal similarity of avian sHSPs could indicate analogous function and substrates. Moreover, based on phylogenetic trees, they can be divided into two groups: one represented by chicken HSP25 and the other by chicken HSP30CL. The similarity between HSP25s versus other sHSPs also showed the same aspect of branching. Thus, avian HSP25 and HSP30CL are distinct sHSPs and may function differently. Furthermore, because of the specific expression of quail and zebra finch *HSP25* and *HSP30CL* in blastoderms at oviposition, and the increased expression of *HSP25* after 7 days of cold storage in both species, which are similar to that in chicken, HSP25 and HSP30CL homologues may be expressed and act in the same way throughout avian species.

Chicken HSP25, induced by various stresses, has been studied in somatic fibroblast cells^19^ and adult tissues^20^,^21^,^22^,^23^. Chicken HSP25 was first isolated by Kawazoe *et al.* Subsequently, Katoh *et al.* observed the accumulation of HSP25 in the aggresomes of somatic fibroblasts. In terms of the genomic structure of chicken *HSP25*, the lack of introns may facilitate rapid expression without disturbance by stressors that could interfere with RNA splicing^34^. Also, *HSP25* was the most significant *sHSP* expressed in testes, brain, liver, and egg muscle of egg-laying and broiler adult chickens in response to acute heat stress exposure^21^,^22^,^23^. Thus, among the *sHSPs* in chicken, *HSP25* could be the first line of the cellular defence against environmental stresses.

Although chicken HSP25 was discovered through accumulation and inclusion formation in chicken somatic cells^19^ and adult tissues^20^,^21^,^22^,^23^, to date, loss of function studies have not been reported. Thus, we next investigated the effects of siRNA-mediated *HSP25* knockdown in chicken blastoderm cells *in vitro* using siRNA-296. Previous studies demonstrated stemness was composed of stress defence, as well as pluripotency in mouse embryonic stem cells. *Hspb1*, a unique signature for stress tolerance, was specific to mouse embryonic stem cells and was reduced along with pluripotency markers by loss of stemness during embryoid bodies formation^5^,^9^. Therefore, we examined pluripotency-related genes, including *NANOG*, *POUV*, *SOX2*, and *CRIPTO*^35^, in chicken blastoderm cells harbouring stemness after *HSP25* knockdown. *In vitro* knockdown of *HSP25* in blastoderm cells caused decreased expression of all pluripotency-related genes, suggesting that *HSP25* seems to have a positive correlation with stem cell property in chickens, like *Hspb1* in mouse embryonic stem cells.

sHSPs are known to be involved in cell survival mechanisms, such as anti-apoptosis^36^,^37^,^38^,^39^,^40^,^41^,^42^, anti-oxidative stress^43^,^44^,^45^,^46^, and autophagy^47^,^48^,^49^. Cellular homeostasis and integrity should be maintained appropriately for future embryonic development. Nevertheless, the role of chicken *HSP25* and dormancy-specific sHSPs related to biological processes in cell protection have remained largely unknown. We investigated blastoderm cells after *HSP25* knockdown followed by mitomycin C treatment to induce apoptosis. Annexin V/PI analysis showed that suppression of *HSP25* decreased live cells significantly and increased apoptotic cells versus the control. Also, based on qRT-PCR analysis, anti-apoptotic genes were downregulated in knockdown blastoderm cells. These results suggest that chicken *HSP25* in blastoderm cells is important for regulating apoptosis against stress. Additionally, we discovered an anti-oxidant effect of *HSP25*. DCFDA and qRT-PCR analysis indicated that knockdown of *HSP25* followed by H_2_O_2_ treatment increased ROS production significantly and downregulated some anti-oxidant genes in blastoderm cells versus the control. Furthermore, we measured live and dead cells using annexin V/PI flow cytometry. Due to increased ROS production after *HSP25* knockdown, live cells were decreased significantly, but not apoptotic cells. Rather, necrotic cells were increased, consistent with a previous study indicating ROS-induce necrosis^50^. Finally, to assess the pro-autophagic function of chicken *HSP25*, we examined autophagy stimulation with the autophagy marker LC3 after inhibition of *HSP25*. Quantification using flow cytometry indicated that autophagic activation was suppressed significantly with *HSP25* knockdown versus the control. Additionally, the relative expression of pro-autophagic genes was downregulated by *HSP25* knockdown. Consequently, chicken HSP25 may be involved in the regulation of processes including apoptosis, anti-oxidative stress, and autophagy for cellular integrity in blastoderm cells.

In conclusion, we found that *HSP25* may be associated with stress-tolerant characteristics in blastoderm cells in chicken. Also, homologues of chicken HSP25 were present in and conserved among avian species. Furthermore, we performed chicken *HSP25* knockdown experiments in blastoderm cells to examine functions associated with apoptosis, anti-oxidative stress, and autophagy. Finally, we demonstrated avian *HSP25* is the first line of cellular defence against environmental stresses and is important in protecting future embryonic cells in the avian blastoderm in cold torpor, as a dormancy state.

## Methods

### Experimental animals and animal care

The care and experimental use of animals was approved by the Institute of Laboratory Animal Resources, Seoul National University (SNU-150827-1). Animals were maintained according to a standard management program at the University Animal Farm, Seoul National University, Korea. The procedures for animal management, reproduction, and embryo manipulation adhered to the standard operating protocols of our laboratory.

### Multiple sequence alignment, pair-wise comparison, and phylogenetic analysis

Amino acid sequences of small heat shock proteins containing the α-crystallin domain HSPB9-like from the NCBI database that were analysed included chicken HSP25, HSP30C-like (NP_001010842, XP_003642880), Japanese quail HSP25, HSP30C-like (XP_015741136, XP_015741135), collared flycatcher HSP30C-like (XP_005059698), Tibetan ground-tit HSP30C-like (XP_005531515), zebra finch HSP25-like, HSP30C-like (NP_001232665, XP_004174712), rock pigeon HSPB11-like, HSP30C (XP_005513649, XP_005513650), African clawed frog HSP30C, HSP30D (NP_001165977, NP_001165976), Atlantic salmon HSP30 (NP_001134440), human HSPB9 (NP_149971), and mouse HSPB9 (NP_083583). For pair-wise comparisons, multiple sequence alignments, and phylogenetic trees, the amino acid sequences of the small heat shock proteins above were aligned using the Geneious software (ver. 6.0.4; Auckland, New Zealand) with default penalties for gaps and the protein weight matrix of BLOSUM (blocks substitution matrix). A phylogenetic tree was reconstructed using a neighbour-joining method.

### Sample preparation

Blastoderms were obtained within 6 h after oviposition from White Leghorn chickens (WL), Japanese quail (JQ), and zebra finch (ZF). WL, JQ, and ZF eggs were incubated with intermittent rocking at 37.5 °C under 60–70% relative humidity. Chicken blastoderm cells were collected by gentle dissociation of EGK stage X blastoderms from WL eggs^25^. WL eggs were stored at 16 °C under 70–80% relative humidity for 2 weeks. The egg-laying times of the WL hens were recorded and intrauterine eggs from EGK stages III-VIII were harvested using an abdominal massage technique^51^. To collect oocytes and zygotes, WL hens were sacrificed and the follicles were collected. Chicken embryonic fibroblasts (CEFs), quail embryonic fibroblasts (QEFs), and zebra finch embryonic fibroblasts (ZEFs) were collected by dissociating the embryonic body of Hamburger and Hamilton (HH) stage 28 in 0.05% trypsin-EDTA (GIBCO Invitrogen, Grand Island, NY, USA) at 37 °C for 10 min^52^. Cells were then cultured in Dulbecco’s modified Eagle’s medium (DMEM; Thermo Fisher Scientific, Inc., Waltham, MA, USA) containing 10% FBS and 1% antibiotic-antimycotic (Invitrogen) in a 5% CO_2_ atmosphere at 37 °C.

### RT-PCR and quantitative real-time PCR analysis

Total RNA was isolated using the Trizol Reagent (Invitrogen, Carlsbad, CA), according to the manufacturer’s protocol. For RT-PCR and quantitative real-time PCR analysis of mRNAs, total RNA (1 μg) was used as template for cDNA synthesis using the SuperScript III First-Strand Synthesis System (Invitrogen). The cDNA was serially diluted five-fold and was equalised quantitatively for PCR amplification. Primers for real-time PCR of each gene transcript were designed using the program Primer3 (Supplementary Table S1; http://frodo.wi.mit.edu/). RT-PCR was performed with an initial incubation at 95 °C for 5 min, followed by 35 cycles of 95 °C for 30 s, 59 °C for 30 s, and 72 °C for 30 s. The reaction was terminated after a final incubation at 72 °C for 5 min. Real-time PCR analysis was performed using a CFX96 real-time PCR detection system with a C1000 thermal cycler (Bio-Rad Laboratories, Hercules, CA, USA). The qRT-PCR conditions were 95 °C for 3 min followed by 40 cycles of 95 °C for 30 s, 59 °C for 30 s, and 72 °C for 30 s. Melting curve profiles were analysed for amplicons. Each test sample was run in triplicate. The relative quantification of gene expression was analysed with the 2^-ΔΔCt^ method^53^.

### Chicken blastoderm cell culture

Stage X blastoderm cells were prepared as described previously^54^. Briefly, blastoderm cells were prepared from the pellucida area of WL embryos at stage X and dissociated mechanically into single cells. Single stage X cells were centrifuged (850 rpm, 3 min) and washed twice in PBS. Blastoderm cells were cultured according to previous descriptions with minor modifications^55^. Cells were cultured in N2B27/2i medium containing DMEM/F-12 (Gibco), Neurobasal (Gibco), 55 mM β-mercaptoethanol (Gibco), 200 mM L-glutamine (Gibco), N2-Supplement (100×, Gibco), and B27 supplement-vitamin A (50×, Gibco). The medium was supplemented with two inhibitors, 3 μM CHIR99021 and 1 μM PD0325901 (Stemgent, San Diego, CA, USA).

### Transfection and chemical treatment of chicken blastoderm cells

*HSP25*-specific siRNAs were designed and purchased from Bioneer Corporation (Supplementary Table S2; Daejeon, Korea). For the transfection of siRNAs into the cultured blastoderm cells, Lipofectamine RNAiMAX (Invitrogen) was used according to the manufacturer’s protocol for 48 h. For the *in vitro* induction of apoptosis, reactive oxygen species (ROS) production, and autophagy, cells were treated with mitomycin C (Sigma-Aldrich, St. Louis, MO, USA), H_2_O_2_ (Sigma), and MG132 (Sigma). Specifically, 0, 30, and 60 μM mitomycin C was added for 4 h; 0, 100, and 200 μM H_2_O_2_ was added for 4 h; and 0, 5, and 10 μM MG132 was added for 24 h. After each treatment, cells were harvested and analysed.

### TUNEL assay

Cells were washed and concentrated on glass slides. After fixation in 2% paraformaldehyde for 15 min, the cells were incubated in a permeabilisation solution (0.1% Triton X-100 in PBS) for 10 min. Apoptotic cells were identified using an *in situ* Cell Death Detection Kit and TMR red (Roche Applied Science, Basel, Switzerland) that stains apoptotic cells red. Cells were counterstained with DAPI, mounted, and analysed under a fluorescence microscope (TU-80, Nikon).

### Flow cytometry

To examine apoptotic cell death, annexin V/propidium iodide (PI) (Thermo Fisher Scientific) staining was performed according to manufacturer’s protocol and cells were analysed by flow cytometry (FACSCalibur, BD Biosciences, San Jose, CA, USA). Annexin V and PI double-negative cells were considered viable, annexin V-positive and PI-negative (early apoptotic) cells and annexin V- and PI-positive (late apoptotic) cells were considered apoptotic, and PI only-positive cells were considered necrotic. To assess cellular ROS, a 2′,7′-dichlorofluorescein diacetate (DCFDA) assay (Abcam, Cambridge, UK) was performed according to the manufacturer’s protocol and analysed with the FACSCalibur. To assay autophagy activity, cells were labelled with anti-light chain 3 (LC3) (Abcam) followed by staining with Alexa488-conjugated goat anti-mouse IgG antibodies. Next, cells were analysed with FACSCalibur and data were analysed with the FlowJo software (ver. 7.6.5; Tree Star, Ashland, OR, USA).

### Statistical analyses

Significant differences between groups were examined statistically using Student’s *t*-test and one-way ANOVA. A *P* value < 0.05 was considered to indicate statistical significance (***P < 0.001, **P < 0.01, and *P < 0.05).

## Additional Information

**How to cite this article**: Hwang, Y. S. *et al.* The avian-specific small heat shock protein HSP25 is a constitutive protector against environmental stresses during blastoderm dormancy. *Sci. Rep.* **6**, 36704; doi: 10.1038/srep36704 (2016).

**Publisher’s note**: Springer Nature remains neutral with regard to jurisdictional claims in published maps and institutional affiliations.

## Supplementary Material

Supplementary Information
