# Supplementary material for: The avian-specific small heat shock protein HSP25 is a constitutive protector against environmental stresses during blastoderm dormancy
Source: Sci Rep. 2016 Nov 9;6:36704. doi: 10.1038/srep36704 (PMC5101479; doi:10.1038/srep36704)
Supplement: Supplementary Information [file srep36704-s1.pdf]

The avian-specific small heat shock protein HSP25 is a constitutive protector against environmental stresses during blastoderm dormancy

Young Sun Hwang[1], Mee Hyun Ko[1], Young Min Kim[1], Young Hyun Park[1], Tamao Ono[2] and Jae Yong Han[1,3,*]

[1]Department of Agricultural Biotechnology and Research Institute of Agriculture and Life Sciences, College of Agriculture and Life Sciences, Seoul National University, Seoul 08826, Korea. [2]Division of Animal Science, Faculty of Agriculture, Shinshu University, Minamiminowa, Nagano 399-4598, Japan. [3]Institute for Biomedical Sciences, Shinshu University, Minamiminowa, Nagano 399-4598, Japan. [*]Correspondence and requests for materials should be addressed to J.Y.H (email: jaehan@snu.ac.kr)

Supplementary Table S1. **Primers used for quantitative real-time PCR**

Accession No.	Gene	Primer sequences	
		Forward (5’→3’)	Reverse (5’→3’)
NM_204305 (Chicken)	*GAPDH*	GGTGGTGCTAAGCGTGTTAT	ACCTCTGTCATCTCTCCACA
XM_015873412 (Quail)	*GAPDH*	GGGAAGTTGTGGAGGGATGG	GGTTGGCACACGGAAAGCCA
NM_001198610 (Zebra finch)	*GAPDH*	GGGAAGTTGTGGAGGGATGG	GGTTGGCACACGGAAAGCCA
NM_205518	*ACTB*	AGGAGATCACAGCCCTGGCA	CAATGGAGGGTCCGGATTCA
NM_001010842 (Chicken)	*HSP25*	AGTTCATGAGCAGCTTCGAG	GTGTTCTGCGTCTCCTTCTG
XM_015885650 (Quail)	*HSP25*	AGTTCATGAGCAGCTTCGAG	GTGTTCTGCGTCTCCTTCTG
NM_001245736 (Zebra finch)	*HSP25*	AGTTCATGAGCAGCTTCGAG	CTTGTAGGAGAAGGAGCCCT
XM_003642832 (Chicken)	*HSP30CL*	GGCAGTTCATGAGCAGCTTC	ACTTGTAGGAGAAGGAGCCC
XM_015885649 (Quail)	*HSP30CL*	TGGAGATGGAGAGAGCTCGG	ACTTGTAGGAGAAGGAGCCC
XM_004174664 (Zebra finch)	*HSP30CL*	TGGAGATGGAGAGAGCTCGG	ACTTGTAGGAGAAGGAGCCC
NM_205290	*HSPB1*	CGTGCCCTTCACCTTCCTCA	TGGGCAGCAGACGGAAGTATC
NM_001001527	*HSPB2*	GGCCTCGGATCAACAAGCAG	TCTCGGGAGATGAAGCCGTG
XM_001231557	*HSPB3*	CTGCCGAGGAGCTGGAAGAA	CTGGGCGAAACTGCACAACA
NM_001030797	*HSPB4*	TTACCATCCAGCACCCCTGG	GAAATGCCCGACTCCAGCAC
NM_205176	*HSPB5 (CRYAB)*	CCAGGGCCATTCAAGAGCAC	CGTGGCTTCCTTGAGCCAAC
XM_427836	*HSPB7*	CCCGGATGCAAACAAGAAGC	TTGGTGACCTTCCAGGTGGG
XM_415280	*HSPB8*	GCGTCAACGTGCACAGCTTC	AGGCGAAGACAGTGATGGGG
XM_418368	*HSPB10*	GCTTTTTGGAGGACCACGAG	GTTTTCTCCGTACACTGGCC
NM_001277613	*HSPB11*	TGGCTTTCCCAAGAGTGTGA	TCACCCTGTGCACTGATACA
NM_001146142	NANOG	AACTCTGCGGGGCTGTCTTG	AAAAGTGGGGCGGTGAGATG
NM_001110178	*POUV*	TGAAGGGAACGCTGGAGAGC	ATGTCACTGGGATGGGCAGAC
NM_205188	*SOX2*	CCCCTTTCATACCCCTCCCT	CGGGCTGTTCTTCTGGTTGTT
NM_204700	*CRIPTO*	CGACACAGCGCAAAAGCAAT	AGCCCTTCATCACCCAGTCG
NM_205339	*BCL2*	CTTTATCCTCCTGCCCCTCG	TTCTTCCGCTTCGTCAGCAA
NM_001025304	*BCL2L1*	TTCAGCGACCTCACCTCCCA	GCCCCCAGTTCACACCATCA
NM_001031215	*CAT*	TCAGAAGCCAGATGCCTTGA	CCTTTCGCATGCACAACTCT

NM_001277853	*GPX1*	CATGTTCGAGAAGTGCGAGG	AAGTTCCAGGAGACGTCGTT
NM_001277854	*GPX2*	CTTCTACGACCTCAGTGCCA	TGAGGCAGTTGAGGATCTCC
NM_001163232	*GPX3*	GAACTCCGAGATCCTCCCTG	GGCTCCCAGAAGAGGTTCTT
XM_003642871	*GPX4*	GAGGTGGCGGGATGGGTT	GTACTGCTCCAGGGACACAT
NM_001163245	*GPX7*	AAGCCTTCCTCTTCCTTCCC	GACAACTAACGACACCGAGC
XM_423834	*GPX8*	GGTTGTAAACGTGGCGAGTT	TTCCCTTTGGCAAAAGCCTC
XM_421555	*PTEN*	CCGTTGGTCAACTTTGCTCA	CATGTCCCCAGGTAACCACT
XM_416328	*DRAM1*	GTGGCCTTTGGTTTCGTCTT	TGCTCCTCTGCAATTCTCCA
NM_001006332	*BECN1*	GCTGAAGCTCGATACCTCCT	ATTCTGGCTGGTGGGATGAA
NM_001030839	*UVRAG*	AAGTACTTGGGGCAGCAGAT	AGCTCTGTGAAGCCTAAGCA
XM_003643073	*ATG12*	AGTTGTCTGCTTGCATGGTG	ATCGGTTCCAGTGCCACTTA
NM_001006409	*ATG5*	GGGCATGTTAACTGCTTGCT	CGGTGAAGAAAAGCAGCAGT
NM_001030592	*ATG7*	ATCAAGAGACACAGGCTGCT	TCATGGCAGTCAGAATGGGT
NM_001031461	*MAP1LC3B*	TGTACGAGAGCGAGAAGGAC	GCGTAGGTGACATCCGTTTC

Supplementary Table S2. **List of *HSP25*-specific siRNA sequences for knockdown experiments**

Accession No.	Gene	Sequences	Start position
NM_001010842	*HSP25*	S 5'-GAGAAGGCUCGGCAGUUCA UU-3'<br>AS 5'-UGAACUGCCGAGCCUUCUC UU-3'	296
		S 5'-CAGAAGGAGACGCAGAACA UU-3'<br>AS 5'-UGUUCUGCGUCUCCUUCUG UU-3'	497
N/A	Non-specific siRNA[#]	S 5'-CCUACGCCACCAAUUUCGU-3'<br>AS 5'-GGAUGCGGUGGUUAAAGCA -3'	N/A

[#] The non-specific siRNA has no complementary sequence in the chicken genome and was used as a control for gene silencing.
